# Are dietary intake parameters of vitamin A, carotene, retinol appropriate factors to evaluate the risk of diabetic retinopathy?

**DOI:** 10.1097/MD.0000000000033969

**Published:** 2023-06-02

**Authors:** Yu-Jin Choi, Jin-Woo Kwon, Donghyun Jee

**Affiliations:** a Department of Ophthalmology and Visual Science, St. Vincent’s Hospital, College of Medicine, Catholic University of Korea, Suwon, Republic of Korea.

**Keywords:** carotene, diabetic retinopathy, Korea, retinol, vitamin A

## Abstract

The purpose of this study is to investigate whether dietary parameters of vitamin A, carotene, and retinol are sufficient for assessing the risk of diabetic retinopathy (DR). This was a population-based cross sectional study using systematic stratified, multilevel, nationwide, clustered sampling methods. From 2008 to 2012, 1948 subjects aged ≥ 40 years who participated in the Korean National Health and Nutrition Examination Survey were included. Participants underwent standardized interviews, dietary vitamin A estimation, carotene and retinol level evaluation, and eye examination. Daily dietary intake was evaluated using data in the form of a single 24-hour recall. The odds ratio (OR) of dietary vitamin A between extreme quartiles for DR was 0.72 (95% confidence interval [CI], 0.45–1.16, *P* for trend = .462) after adjusting for covariates such as age, sex, hypertension, hemoglobin A1c levels, and diabetes duration. The adjusted OR of dietary carotene between extreme quartiles for DR was 0.65 (95% CI, 0.39–1.09, *P* for trend = .220). The adjusted OR of dietary retinol between extreme quartiles for DR was 1.05 (95% CI, 0.62–1.80, *P* for trend = .279). There was no statistical significance in proliferative DR and Vision-threatening Dr Our study did not find evidence that the risk of DR is correlated with dietary vitamin A levels. Dietary intake parameters of vitamin A, carotene, and retinol might be insufficient to determine the association between the risk of Dr To demonstrate an association for the risk of DR, the use of serum information and not dietary information is needed.

## 1. Introduction

Diabetes is one of the chronic diseases that are associated with various microvascular complications.^[1]^ Diabetic retinopathy (DR), which is one of diabetes complications, leads blindness in adults worldwide.^[2,3]^ We previously reported that DR was observed in 15.8% of diabetic patients, of which 4.6% were at risk of blindness requiring treatment for representative Korean population.^[4]^ As the prevalence of diabetes increases worldwide, interest in risk factors for DR is increasing.^[5]^ Poorer blood pressure and glycemic control and longer disease duration are strongly associated with risk of DR.^[3]^ Persistent hyperglycemia can activate metabolic pathways and increase oxidative stress. Previous studies have demonstrated importance for oxidative stress in pathogenesis of DR.^[6,7]^ This suggests that the administration of antioxidants can prevent the development of oxidative stress and DR in diabetic patients.

Vitamin A is a fat-soluble vitamin, antioxidant and is an important nutrient in the regulation of glucose and lipid metabolism.^[8]^ It is essential for embryonic development and growth, maintaining the immune system, and forming rhodopsin, a light-absorbing molecule necessary for both color vision and scotopic vision, in combination with protein opsin.^[9]^ Vitamin A occurs as 2 principal forms, retinol and carotene in foods. Retinol in the body works by converting it to retinal and retinoic acid. Retinol conducts an important part in visual system by conversion between all-trans-retinal and 11-cis-retinal.^[10]^ Retinol is mainly found in animal food and is contained in fish, dairy products, and meat.^[11]^ Carotene is an antioxidant that decreases the levels of the free-radicals that cause DNA damage.^[12]^ It is an unsaturated hydrocarbon substance synthesized by plants and is an important photosynthetic pigment for photosynthesis. Carotene scatters orange or red light, and yellow light. Carotene is found in fruits with orange, yellow and vegetables. Carotene is contained in carrot, tomato, red bell pepper, kale, mangoes, and broccoli.^[13,14]^

As life expectancy increases, people’s interest in nutrients is increasing for the quality of life. Interest in research on the relationship between nutrients and ophthalmic diseases is also increasing. Previous studies on the association between DR and vitamin A have been performed in the Australian and Chinese regions.^[15–17]^ However, studies for the relationship between DR and vitamin A have been limited. Also, some studies suggest that vitamin A is associated with diabetic retinopathy and some studies do not.^[15–20]^ We think that methodology is important in conducting research. The results may vary according to the form of data (nutrient intake levels or serum levels). Thus, this study was conducted to investigate whether dietary parameter of vitamin A, carotene and retinol is sufficient to assess risk of Dr

## 2. Methods

### 2.1. Study population

This study was based on data from Korean National Health and Nutrition Examination Survey (KNHANES) (2008–2009) and KNHANES (2010–2012), the 4th and 5th national representative survey administrated by the Korea Center. The KNHANES is a population-based cross-sectional study using systematic stratified, multi-level, nationwide, clustered sampling methods with proportional allocations based on the National Census Register of the noninstitutional civilian population of Korea. The KNHANES consists of a nutrition survey, a health examination survey and a health interview survey.^[21]^

**Figure 1.**
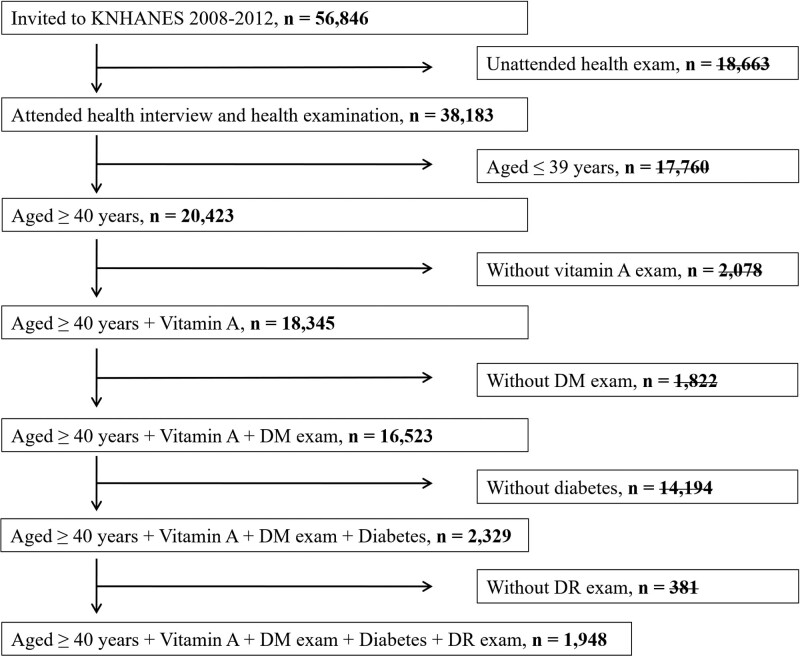
Flow chart presenting the selection of study participants.

The study population comprised 56,846 individuals who responded to the 4th and 5th KNHANES. Of these, 18,663 subjects who had not been attended health exam and 17,760 subjects aged ≤ 39 years were excluded. Dietary vitamin A exam and diabetes mellitus exam were not mandatory for everyone who participated in the KNHANES survey. Of 20,423 subjects, 2078 who had not been attended dietary vitamin A exam and 1822 who had not been attended diabetes mellitus exam were excluded. The 381 subjects who did not take a fundus photograph were excluded, of the 2329 subjects with diabetes, Finally, 1948 subjects aged 40 years or older were eligible for the analysis (Fig. 1). The study was approved by the Institutional Review Board of the Catholic University, Korea (IRB number: VC22ZESI0175). This study followed the principles of the Helsinki Declaration for research.

### 2.2. Demographic characteristics

The health interview and general questionnaire were used to collect the general characteristics of participants, including sex, age, body mass index, exposure time to the sun, smoking status. Data for sunlight exposure time were acquired by selecting 2 options: ≥5 hours or < 5 hours for a day. Smoking status was sorted in 3 categories: never smokers, past-smokers and current smokers.

Blood samples were taken after fasting for 10 to 12 hours. The hemoglobin A1c, fasting glucose, triglyceride and total cholesterol were checked by Hitachi automatic analyzer 7600 (Hitachi, Ltd., Tokyo, Japan). All samples were refrigerated immediately, and transported to the Ministry of Health and Welfare-certified science lab.

Blood pressure was checked by a mercury sphygmomanometer. After 3 measurements at interval of 5 minutes, the average of measurements was used for analysis. Hypertension was defined as having systolic blood pressure >140 mm Hg, diastolic blood pressure >90 mm Hg, or using prescribed hypertension treatments. Prehypertension was defined as a systolic pressure from 120 to 139 mm Hg or a diastolic pressure from 80 to 89 mm Hg.^[22]^

### 2.3. Estimation assessment for dietary intake of vitamin A

Daily dietary intake was evaluated in the form of a single 24-hour recall recorded in KNHANES. A 24-hour food recall is a well-established method of assessing diet used. This technique quantifies the amount of food or drink consumed during the 24-hour period prior to the interview or throughout the day, from the first meal in the morning to the last food or drink at night. Participants reported all dietary foods that they ate the previous day through interviews. The quantity of nutrient intake was assessed with a 24-hour food recall conducted in person by trained nutritionists. In this study nutrients including vitamin A, carotene and retinol were estimated. Nutrient intakes including vitamin A, carotene and retinol were assessed with a questionnaire administered. It was calculated using the frequency of intake per day, the ratio to the amount based on intake, and the nutrient content for each item. Data from the recall was converted into estimated quantities of nutrients and vitamins based on the Food Composition Table developed by the National Rural Resources Development Institute (7th revision) using a web-based statistical analysis system program related to nutrition and food intake frequency survey.^[23]^ The sum of nutrient intake from all foods and beverages consumed by an individual during the day was calculated. The quantity of dietary intake of vitamin A, carotene and retinol was calculated through food intake frequency surveys.

### 2.4. Assessment of diabetes and diabetic retinopathy

Diabetes diagnosis was assigned to those who were receiving drug medication as hypoglycemic agent, or those with fasting blood glucose > 126 mg/dL, random blood glucose > 200 mg/dL or HbA1c > 6.5.^[24]^ In participants with diabetes, 7 standard field photographs were conducted after pharmacological pupil dilatation by using fundus photography (TRC-NW6S; Topcon, Tokyo, Japan). DR was diagnosed if any lesion defined by the Early Treatment for Diabetic Retinopathy Study severity scale was present: these was characteristic lesion as hemorrhage, microaneurysm, hard exudate, intraretinal microvascular abnormalities, cotton wool spots, new vessels, venous beading and cotton wool spots. DR severity score was assessed using a modified version of the Airlie House Classification system. The DR severity was categorized into 3 groups according to the following criteria: Any DR (a level > 14), proliferative diabetic retinopathy (PDR) (a level > 60), Vision-threatening diabetic retinopathy (VTDR) (the presence of severe non proliferative DR, PDR, or clinically significant macular edema).^[25,26]^

### 2.5. Statistical analysis

A complex sampling design was used for the analysis. Strata, sampling units, and sampling weights were used to obtain unbiased point estimates and linearized standard errors. We analyzed the crude weighted mean and standard error for continuous variables and percentages and standard errors for categorical variables according to the presence of Dr We did not indicate unweighted number on the table because of complex sampling design.

Logistic regression analysis were used to evaluate association between dietary vitamin A, carotene, retinol level and Dr The value that is not adjusted is defined as “Crude.” “Model 1” was analyzed after adjusting age and sex. For “Model 2,” we adjusted for age, sex and other confounding factors, including the hypertension, hemoglobin A1c levels and diabetes duration which have been verified in previous studies as independent risk factors.^[3,4]^ The statistical analysis was conducted by SPSS (ver. 18.0; SPSS, Inc., Chicago, IL). *P* values < .05 was regarded as statistical significance.

## 3. Results

The demographic characteristics of 1948 subjects were showed according to DR status (Table 1). Participants with DR were more likely to have an old age (*P* = .010), higher systolic blood pressure (*P* = .015), lower diastolic blood pressure (*P* = .001), longer diabetes duration (*P* < .001), higher hemoglobin A1c (*P* < .001), higher fasting glucose (*P* < .001), higher hyperlipidemia level (*P* = .034) compared to those without Dr And there was a difference in DR status according to body mass index (*P* = .001).

**Table 1:** Demographic characteristics according to DR status. We did not indicate unweighted number on the table because of complex sampling design.

Characteristics	Without DR	Any DR	*p*	Participants
Male (%)	54.3 (1.4)	53.3 (3.3)	.771	45.9 (1.3)
Age (yr)	59.5 (0.3)	61.4 (0.6)	.010	60.4 (0.3)
Systolic blood pressure (mm Hg)	127.5 (0.5)	130.9 (1.2)	.015	129.2 (0.6)
Diastolic blood pressure (mm Hg)	78.6 (0.3)	76.0 (0.7)	.001	77.3 (0.4)
Diabetic duration (years)	6.9 (0.2)	11.2 (0.4)	<.001	9.0 (0.2)
HbA1c (%)	7.19 (0.0)	8.04 (0.1)	<.001	7.61 (0.0)
Fasting glucose (mg/dL)	139.5 (1.2)	157.4 (2.9)	<.001	148.5 (1.5)
Total cholesterol (mg/dL)	188.5 (1.2)	185.7 (2.6)	.336	187.1 (1.4)
Triglyceride (mg/dL)	187.4 (6.3)	181.0 (8.0)	.545	184.2 (4.9)
Vitamin A (µgRE)	782.5 (33.4)	885.0 (134.5)	.539	823.7 (71.3)
Vitamin A (%)			.367	
Vit A < 285.8	26.6 (1.4)	31.6 (3.0)		27.5 (1.3)
285.8 < Vit A < 557.3	25.3 (1.4)	25.7 (2.9)		25.4 (1.3)
557.3 < Vit A < 994.1	23.7 (1.3)	20.2 (2.4)		23.1 (1.1)
Vita A > 994.1	24.4 (1.5)	22.5 (2.9)		24.1 (1.4)
Carotene (µg)	4121.2 (163.4)	4687.8 (787.4)	.466	4404.5 (415.0)
Carotene (%)			.137	
Carotene < 1391.8	26.4 (1.4)	34.0 (3.3)		27.7 (1.3)
1391.8 < Carotene < 2905.9	26.4 (1.4)	24.2 (2.8)		26.0 (1.2)
2905.9 < Carotene < 5306.0	23.4 (1.4)	19.8 (2.4)		22.8 (1.2)
Carotene > 5306.0	23.8 (1.5)	21.9 (2.8)		23.5 (1.3)
Retinol (µg)	88.1 (19.9)	76.7 (14.5)	.652	82.4 (12.1)
Retinol (%)			.641	
Retinol < 5.8	29.2 (1.5)	26.8 (3.2)		28.8 (1.4)
5.8 < Retinol < 32.4	23.2 (1.3)	27.0 (2.8)		23.9 (1.2)
32.4 < Retinol < 93.3	24.2 (1.5)	24.1 (2.7)		24.2 (1.4)
Retinol > 93.3	23.4 (1.4)	22.2 (2.8)		23.2 (1.2)
Hypertension (%)			.356	
Normal	17.0 (1.1)	16.9 (2.3)		17.0 (1.0)
Prehypertension	22.7 (1.3)	26.7 (2.8)		23.4 (1.2)
Hypertension	60.3 (1.5)	56.4 (2.9)		59.6 (1.4)
Body mass index (kg/m^2^)			.001	
BMI < 18.5	0.9 (0.3)	2.4 (1.0)		1.1 (0.3)
18.5 ≤ BMI < 25	49.0 (1.5)	59.8 (3.2)		50.8 (1.4)
BMI ≥ 25	50.1 (1.5)	37.8 (3.2)		48.0 (1.4)
Hyperlipidemia (%)	26.1 (1.2)	32.8 (3.1)	.034	27.2 (1.2)
Hypertriglyceridemia (%)	29.4 (1.5)	29.6 (3.3)	.953	29.4 (1.3)
Sun exposure (≥5hr/d, %)	23.1 (1.6)	19.2 (2.4)	.168	22.5 (1.4)
Smoking status			.271	
Never (%)	48.6 (1.5)	49.0 (3.3)		48.7 (1.4)
Former (%)	19.1 (1.2)	23.0 (2.9)		19.8 (1.1)
Current (%)	32.3 (1.4)	27.9 (2.8)		31.6 (1.2)

Values are written as weighted frequency (%) or weighted means with standard error.

BMI = body mass index, DR = diabetic retinopathy.

The dietary vitamin A intake levels were associated with any DR, PDR and VTDR in crude value. The odds ratios (ORs) in crude value for any DR, PDR and VTDR were 0.51 (95% confidence interval [CI], 0.35–0.73, *P* for trend < .001), 0.40 (95% CI, 0.18–0.88, *P* for trend = .046), and 0.41 (95% CI, 0.21–0.80, *P* for trend = .008), respectively, for participants in the highest dietary vitamin A quartile relative to those in the lowest quartile and they were statistically significant (Table 2). However, these associations became insignificant after adjusting confounding factors including age, sex, hypertension, hemoglobin A1c levels and diabetes duration.

**Table 2:** Prevalence rates and adjusted ORs of any DR, PDR, VTDR, stratified regarding quartile categories of dietary vitamin A in adults who represent Korea over the age of 40. We didn’t indicate unweighted number on the table because of complex sampling design. Prevalence rates were signified as weighted estimates [%] (standard error [%], 95% CI).

Quartile vitamin A level (µgRE)	Prevalence	Crude	Model 1	Model 2
Any DR				
Quartile 1 (<285.8)	4.7 (0.5, 3.8–5.9)	1.0 (reference)	1.0 (reference)	1.0 (reference)
Quartile 2 (285.8–557.3)	3.2 (0.4, 2.5–4.1)	0.66 (0.47–0.92)	0.79 (0.56–1.11)	0.85 (0.56–1.31)
Quartile 3 (557.3–994.1)	2.4 (0.3, 1.8–3.0)	0.48 (0.34–0.68)	0.62 (0.43–0.89)	0.72 (0.45–1.15)
Quartile 4 (>994.1)	2.5 (0.3, 1.9–3.3)	0.51 (0.35–0.73)	0.67 (0.45–0.99)	0.72 (0.45–1.16)
*P* for trend	<.001	<.001	.062	.462
PDR				
Quartile 1 (<285.8)	0.9 (0.2, 0.5–1.4)	1.0 (reference)	1.0 (reference)	1.0 (reference)
Quartile 2 (285.8–557.3)	0.3 (0.1, 0.2–0.6)	0.36 (0.15–0.83)	0.45 (0.19–1.05)	0.49 (0.19–1.26)
Quartile 3 (557.3–994.1)	0.4 (0.1, 0.2–0.7)	0.50 (0.24–1.02)	0.69 (0.32–1.49)	0.81 (0.31–2.05)
Quartile 4 (>994.1)	0.4 (0.1, 0.2–0.7)	0.40 (0.18–0.88)	0.57 (0.24–1.32)	0.46 (0.17–1.26)
*P* for trend	.020	.046	.290	.320
VTDR				
Quartile 1 (<285.8)	1.4 (0.3, 1.0–2.0)	1.0 (reference)	1.0 (reference)	1.0 (reference)
Quartile 2 (285.8–557.3)	0.6 (0.2, 0.3–1.0)	0.39 (0.20–0.78)	0.46 (0.23–0.93)	0.43 (0.19–0.95)
Quartile 3 (557.3–994.1)	0.7 (0.1. 0.5–1.1)	0.51 (0.29–0.89)	0.64 (0.35–1.16)	0.73 (0.35–1.52)
Quartile 4 (>994.1)	0.6 (0.2. 0.3–1.0)	0.41 (0.21–0.80)	0.53 (0.26–1.07)	0.49 (0.20–1.20)
*P* for trend	.007	.008	.114	.150

Model 1: adjusted for age and sex. Model 2: adjusted for age, sex, hypertension, hemoglobin A1c levels and diabetes duration.

CI = confidence interval, DR = diabetic retinopathy, OR = odds ratio, PDR = proliferative diabetic retinopathy, VTDR = Vision-threatening diabetic retinopathy.

The dietary carotene intake levels were associated with any DR and VTDR in crude value. The ORs in crude value for any DR were 0.62 (95% CI, 0.45–0.87), 0.46 (95% CI, 0.32–0.65), and 0.50 (95% CI, 0.34–0.72), respectively, for participants in the dietary carotene quartile 2, 3, 4 relative to those in the lowest quartile (*P* for trend < .001). Associations between VTDR and the dietary carotene quartiles in crude value showed lower OR trend for participants in the dietary carotene quartile 3 (OR 0.52, 95% CI, 0.28–0.97, *P* for trend = .121) (Table 3). However, these associations became insignificant after adjusting confounding factors including age, sex, hypertension, hemoglobin A1c levels and diabetes duration.

**Table 3:** Prevalence rates and adjusted ORs of any DR, PDR, VTDR, stratified regarding quartile categories of dietary carotene in adults who represent Korea over the age of 40. We didn’t indicate unweighted number on the table because of complex sampling design. Prevalence rates were signified as weighted estimates [%] (standard error [%], 95% CI).

Quartile carotene level (µg)	Prevalence	Crude	Model 1	Model 2
Any DR				
Quartile 1 (<1391.8)	4.8 (0.5, 3.9–6.0)	1.0 (reference)	1.0 (reference)	1.0 (reference)
Quartile 2 (1391.8–2905.9)	3.1 (0.4, 2.4–4.0)	0.62 (0.45–0.87)	0.73 (0.52–1.03)	0.69 (0.43–1.08)
Quartile 3 (2905.9–5306.0)	2.3 (0.3, 1.8–2.9)	0.46 (0.32–0.65)	0.57 (0.40–0.82)	0.65 (0.41–1.03)
Quartile 4 (>5306.0)	2.5 (0.3, 1.9–3.3)	0.50 (0.34–0.72)	0.63 (0.43–0.93)	0.65 (0.39–1.09)
*P* for trend	<.001	<.001	.018	.220
PDR				
Quartile 1 (<1391.8)	0.7 (0.2, 0.4–1.2)	1.0 (reference)	1.0 (reference)	1.0 (reference)
Quartile 2 (1391.8–2905.9)	0.5 (0.1, 0.3–0.9)	0.74 (0.33–1.64)	0.92 (0.40–2.11)	1.08 (0.41–2.84)
Quartile 3 (2905.9–5306.0)	0.4 (0.1, 0.2–0.6)	0.53 (0.24–1.19)	0.72 (0.30–1.72)	1.00 (0.35–2.81)
Quartile 4 (>5306.0)	0.4 (0.1, 0.2–0.7)	0.61 (0.28–1.34)	0.86 (0.36–2.01)	0.87 (0.31–2.40)
*P* for trend	.420	.452	.900	.976
VTDR				
Quartile 1 (<1391.8)	1.2 (0.2, 0.8–1.7)	1.0 (reference)	1.0 (reference)	1.0 (reference)
Quartile 2 (1391.8–2905.9)	0.8 (0.2, 0.5–1.2)	0.64 (0.34–1.19)	0.74 (0.39–1.42)	0.76 (0.35–1.66)
Quartile 3 (2905.9–5306.0)	0.6 (0.1, 0.4–1.0)	0.52 (0.28–0.97)	0.64 (0.33–1.24)	0.84 (0.38–1.83)
Quartile 4 (>5306.0)	0.6 (0.2, 0.4–1.1)	0.55 (0.29–1.03)	0.69 (0.35–1.36)	0.76 (0.33–1.72)
*P* for trend	.139	.121	.560	.884

Model 1: adjusted for age and sex. Model 2: adjusted for age, sex, hypertension, hemoglobin A1c levels and diabetes duration.

CI = confidence interval, DR = diabetic retinopathy, OR = odds ratio, PDR = proliferative diabetic retinopathy, VTDR = Vision-threatening diabetic retinopathy.

The dietary retinol intake levels were associated with any DR in crude value. The ORs in crude value for any DR were 0.57 (95% CI, 0.40–0.83), 0.49 (95% CI, 0.34–0.72), respectively, for participants in the dietary retinol quartile 3, 4 relatives to those in the lowest quartile (*P* for trend = .001) (Table 4). But, these associations became insignificant after adjusting confounding factors including age, sex, hypertension, hemoglobin A1c levels and diabetes duration.

**Table 4:** Prevalence rates and adjusted ORs of any DR, PDR, VTDR, stratified regarding quartile categories of dietary retinol in adults who represent Korea over the age of 40. We didn’t indicate unweighted number on the table because of complex sampling design. Prevalence rates were signified as weighted estimates [%] (standard error [%], 95% CI).

Quartile retinol level (µg)	Prevalence	Crude	Model 1	Model 2
Any DR				
Quartile 1 (<5.8)	4.5 (0.6, 3.5–5.7)	1.0 (reference)	1.0 (reference)	1.0 (reference)
Quartile 2 (5.8–32.4)	3.5 (0.4, 2.8–4.4)	0.78 (0.54–1.11)	0.87 (0.60–1.26)	1.48 (0.90–2.43)
Quartile 3 (32.4–93.3)	2.6 (0.3, 2.1–3.4)	0.57 (0.40–0.83)	0.75 (0.51–1.10)	1.01 (0.63–1.63)
Quartile 4 (>93.3)	2.3 (0.3, 1.7–3.0)	0.49 (0.34–0.72)	0.69 (0.45–1.06)	1.05 (0.62–1.80)
*P* for trend	.001	.001	.318	.279
PDR				
Quartile 1 (<5.8)	0.6 (0.1, 0.4–1.0)	1.0 (reference)	1.0 (reference)	1.0 (reference)
Quartile 2 (5.8–32.4)	0.7 (0.2, 0.4–1.2)	1.21 (0.59–2.46)	1.41 (0.10–2.84)	2.58 (1.11–6.03)
Quartile 3 (32.4–93.3)	0.4 (0.1, 0.2–0.7)	0.62 (0.29–1.34)	0.88 (0.40–1.92)	0.82 (0.31–2.14)
Quartile 4 (>93.3)	0.3 (0.1, 0.2–0.6)	0.52 (0.22–1.19)	0.81 (0.36–1.78)	1.02 (0.38–2.73)
*P* for trend	.115	.166	.552	.054
VTDR				
Quartile 1 (<5.8)	0.9 (0.2, 0.6–1.4)	1.0 (reference)	1.0 (reference)	1.0 (reference)
Quartile 2 (5.8–32.4)	0.9 (0.2, 0.6–1.4)	0.96 (0.52–1.76)	1.08 (0.58–1.99)	1.18 (0.85–3.82)
Quartile 3 (32.4–93.3)	0.8 (0.2, 0.5–1.2)	0.82 (0.43–1.54)	1.08 (0.56–2.08)	1.17 (0.53–2.59)
Quartile 4 (>93.3)	0.6 (0.2, 0.3–1.0)	0.59 (0.29–1.17)	0.83 (0.41–1.69)	1.19 (0.52–2.74)
*P* for trend	.408	.464	.854	.464

Model 1: adjusted for age and sex. Model 2: adjusted for age, sex, hypertension, hemoglobin A1c levels and diabetes duration.

CI = confidence interval, DR = diabetic retinopathy, OR = odds ratio, PDR = proliferative diabetic retinopathy, VTDR = Vision-threatening diabetic retinopathy.

The DR prevalence rates regarding the dietary vitamin A quartiles decreased in quartile 2, 3, 4 relative to the first quartile and these differences were statistically significant for any DR, PDR, and VTDR (*P* for trend < .001, *P* for trend = .020, *P* for trend = .007) (Table 2). The any DR prevalence rates regarding the dietary carotene quartiles were statistically significantly reduced in quartile 2, 3, and 4 relative to the first quartile (*P* for trend < .001) (Table 3). The any DR prevalence rates according to the dietary retinol quartiles decreased statistically significantly in quartile 2, 3, and 4 relative to the first quartile (*P* for trend = .001) (Table 4).

## 4. Discussion

Our study did not find evidence that the risk of any DR, PDR and VTDR was correlated in people with high dietary vitamin A levels compared to those with the lowest dietary vitamin A levels. We further analyzed dietary carotene as a vegetable vitamin and dietary retinol as an animal vitamin, but we did not find evidence for the risk of DR through dietary information.

The reasons for results that vitamin A, carotene, and retinol were not related to the risk of DR is thought to be due to characteristics of variable. First, we evaluated dietary intake for vitamin A, carotene, and retinol. Dietary intake is absorbed into the blood and changes occur when it actually acts on the retina. Considering that vitamin A is a fat-soluble vitamin, the absorption rate may vary if there are diseases that damage the digestion and absorption of fat, such as small intestine abnormalities, chronic digestive disorders, and pancreatic dysfunctions.^[27]^ So, it seems more accurate to measure blood vitamin A levels than dietary vitamin A levels. Moreover, dietary vitamin A acts as form of the retinal in retina.^[11]^ Second, our nutrient data was drawn from a single 24-hour recall survey, which may present an estimate. By the KNHANES investigation, variations between 1 day and 2 to 10 days of 24-hour nutrient recall were not much different. However, the 24-hour recall method may be biased when measuring micronutrients such as dietary vitamin A. As time passes, the food taken the previous day may no longer reflect the average dietary vitamin A intake. Third, 3 important confounding factors that were known to have a significant impact, particularly on diabetic retinopathy, were adjusted in our study.^[15,18]^ In the previous other studies that vitamin A reduces DR, confounding variables were often less adjusted unlike our study. They did not adjust HbA1c, diabetes duration and hypertension

Several studies have been conducted on vitamin A levels and Dr Rostamkhani H et al^[19]^ reported results of increasingly low vitamin levels in the blood according to the severity of DR, with vitamin A levels measured 47.3µg/dL in the group without DR, 42.3µg/dL in the group with nonproliferative DR, and 36.5µg/dL in the group with PDR. Zhang C et al^[15]^ study showed that dietary intake of retinol in the group with DR was significantly lower than the group without DR, and higher dietary retinol intake was associated with 12% lower risk of DR after adjusting potential confounding factors. She C et al reported that the level of alpha carotene in the blood had the effect of lowering the risk of DR by 59% in nonsmokers, and the level of beta carotene in the blood showed no statistical significance.^[18]^ Thus, there is controversy about the relationship between vitamin A and diabetic retinopathy. In our study, there was no statistical difference in dietary vitamin A levels between the DR group and in the group without Dr we also analyzed carotene, which is a vegetable vitamin A, and retinol, an animal vitamin A. In our study, the lowest dietary carotene intake and the lowest dietary retinol intake were associated with higher risk of DR in crude, but after adjusting for potential confounding factors, the dietary carotene intake and the dietary retinol intake were not statistically associated with risk of diabetic retinopathy. This difference of results is thought to be due to the difference in the degree of adjustment of confounding variables between our study and other studies. We adjusted age, sex, diabetes duration, and HbA1c and hypertension, while Zhang C et al^[15]^ did not adjust diabetes duration, HbA1c and hypertension which are known to have a profound effect on Dr She C et al^[18]^ did not adjust hypertension which is important factor for Dr In addition, their study analyzed blood levels which is more accurate, while we analyzed dietary intake. We think that it is critical reason for the difference between our study and previous ones.

We need to be careful when interpreting the results of this study. It may be a misinterpretation to conclude that there is no relationship between vitamin A and diabetic retinopathy. It is reasonable to interpret that our study did not find evidence for association with DR in using dietary vitamin A assessment. There may be bias in the 24-hour recall method and dietary assessment is not as accurate as measuring blood levels. The most studies showing an association between vitamin A and DR used serum vitamin A. We conducted further studies evaluating the relationship between blood vitamin A and DR in subjects of 11,727, and we found that DR and blood vitamin A had an inverse relationship. The OR for any DR after adjusting confounding factors were 0.3 for all diabetes patients. Especially, the OR for any DR was 0.4 in women, and the OR for any DR was 0.1 in patients over 60 years old after adjusting confounding factors (data was not shown, in preparing this result as article). These results support that dietary assessment of vitamin A has limitation to find association between vitamin A and Dr

The major strengths of our study are the relatively large number of participants and the study design that incorporated systemically stratified, multistage, clustered random sampling methods. Moreover, we used KNHANES data of national representative survey that it is a population-based study of Korean adults. Another strength is that our study adjusted HbA1c, hypertension, duration of diabetes, 3 major risk factors for DR, confounding variables which have been proven statistically as important risk factors in previous reports.^[3,4]^ Our study also has several limitations. First limitation is that we designed a cross sectional method for observational nature that incurs difficulties when deducing causality. Second limitation is that selection bias could have been arisen about excluding non-examination participants, because dietary vitamin A exam and diabetes mellitus exam were not mandatory for everyone who participated in the KNHANES survey. Third limitation is that our nutrient data was drawn from a single 24-hour recall survey, which may be biased and present an estimate. Although it has the advantage of knowing dietary information in detail, because it is investigated through the most recent memory, bias may exist because dietary conditions before 24 hours are not reflected. The 4th limitation is that we analyzed the intake of dietary vitamin A, so the amount of serum vitamin A and the change in blood condition after absorption are unknown. Dietary intake was insufficient to determine the exact relationship between vitamin A and Dr Thus, we are currently further studying the relationship between vitamin A status in the blood and DR to solve this limitation.

## 5. Conclusion

The present study provides that dietary intake parameters of vitamin A, carotene and retinol are insufficient to find out association for risk of Dr To demonstrate association for the risk of DR, the using serum information not dietary information is needed. We did not find epidemiologic evidence that intake of dietary vitamin A, carotene, and retinol may be correlated with the risk of any DR, PDR, and VTDR in adults. Further longitudinal studies using serum vitamin A levels are needed before a definitive conclusion on this issue can be drawn.

## Acknowledgements

The authors thank the Epidemiologic Survey Committee of the Korean Ophthalmologic Society for supplying data for this study.

## Author contributions

**Conceptualization:** Yu-Jin Choi, Jin-Woo Kwon, Donghyun Jee.

**Formal analysis:** Donghyun Jee.

**Methodology:** Yu-Jin Choi, Donghyun Jee.

**Supervision:** Donghyun Jee.

**Validation:** Jin-Woo Kwon.

**Writing – original draft:** Yu-Jin Choi.

**Writing – review & editing:** Yu-Jin Choi, Jin-Woo Kwon, Donghyun Jee.
